# Sexual activity and perceived health among Finnish middle-aged women

**DOI:** 10.1186/1477-7525-4-29

**Published:** 2006-05-10

**Authors:** Ansa Ojanlatva, Juha Mäkinen, Hans Helenius, Katariina Korkeila, Jari Sundell, Päivi Rautava

**Affiliations:** 1 Department of Teacher Education, University of Turku, Turku, Finland; 2 Institute of Biomedicine, Center for Reproductive and Developmental Medicine, University of Turku, Turku, Finland; 3 Turku City Hospital, Turku, Finland; 4 Department of Obstetrics and Gynaecology, University of Turku, Turku, Finland; 5 Department of Biostatistics, University of Turku, Turku, Finland; 6 Department of Family Medicine, University of Turku, Turku, Finland; 7 Department of Public Health, University of Turku, Turku, Finland; 8 National Public Health Institute, Helsinki, Finland

## Abstract

**Background:**

An increasing awareness of the need to address sexual and orgasm experiences as part of life quality and an understanding of the great individual differences between women play roles in women's health and medical care across the specialities. Information is lacking as to how negative attitude toward self (NATS) and performance impairment (PI) are associated with sexual activity of middle-aged women. We examined the associations of sexual experience, orgasm experience, and lack of sexual desire with perceived health and potential explanatory variables of NATS and PI.

**Methods:**

Questionnaire was mailed to 2 population-based random samples of menopausal or soon-to-be menopausal women (n = 5510, 70% response) stratified according to age (42–46 and 52–56 years). In multivariate analyses of the associations with the outcome variables, perceived health, NATS, and PI were used as covariates in 6 models in which exercise, menstrual symptoms, and illness indicators were taken into account as well.

**Results:**

Sexual activity variables were associated with perceived health. When present, NATS formed associations with sexual and orgasm experiences, whereas strenuous exercise formed associations with orgasm among 42–46-year-old women alone. Strenuous exercise was not associated with orgasm experience among older women.

**Conclusion:**

NATS and PI are closely tied to orgasm experiences and the meaning of the roles needs to be exposed. Sexual activity deserves to be addressed more actively in patient contact at least with perimenopausal women.

## Background

Impact of menopause on health or sexuality is still imprecise. Appropriate questions [1] have either not been asked or their outcomes are unclear [2]. Women's health has in part been connected with reproduction and gynaecological issues [3], and many practicing physicians believe that the period at or following menopause is associated with health-related problems [1,4] and with less sexual activity than before [5,6]. As an indirect example of health and wellbeing, a recent biological finding linked a woman's long late-life period after menopause with an increased number of offspring [7]. Other similar social indicators are expected with health, sexual, and reproductive issues in the future.

Perceived health status is an indicator of general health and life quality. High education and high household income have presented themselves as indicators of good health [8]. Some ambiguity is present in the findings, however. Menopausal women reported fewer problems and ill health than expected in one study: 80% of the women did not to report depression or 60% did not report hot flushes [1]. In another, 95% of otherwise productive 52–56-year-old and up to 64% of 42–46-year-old women reported that they suffered from mild, moderate, or severe climacteric symptoms [9]. Although 34% of Finnish women reported good perceived health in 1972, 51% in 1981, and 60% in 1992, health is expected to get systematically worse with age [10]. Poor economic life situation and unemployment significantly reduced the mental health status [11].

Many menopausal or soon-to-be menopausal women continue to perceive their health to be good, take care of themselves, and live active and vigorous lives [1]. Women with higher education, regular exercise, and spare-time activities seem to feel better and have fewer complaints than those having less education, infrequent exercise, and no spare-time activities [6]. Further, 45–55-year-old women reported better health when they had experienced a nonterm pregnancy, were in fulltime employment, were separated or divorced, exercised more than once a week, engaged in swimming, and believed that menopausal women worry about losing their minds [12].

Libido, or the frequency of sexual activities, was not seriously affected by the late perimenopausal period in a study on menopausal transition in a population-based sample of 45–55-year old women (n = 2001), whereas more decline was recorded in three sexual issues (sexual responsivity, total score of sexual functioning, woman's positive feelings towards her partner) by the postmenopausal period [12]. Women's sexual activities tend to occur within the context of a relationship [13], and many issues influence them [14]. Frequency of sexual intercourse appears to decrease with age but many Finnish women have let the researchers believe that climacterium rather than age would be to blame [1]. On the other hand, many sexual experiences are defined and studied using male-dominated paradigms [15]. And older individuals are thought to be sexually abstinent when they have medical problems or do not have a partner [13], which may or may not be the case.

Having an orgasm may be considered a powerful demonstration of a person's health status. For instance, an inverse relationship was evident between orgasm frequency and mortality among men [16], but the same is not known about women. More than 2/3 of men (75%) but less than 1/3 of women (29%) always achieved orgasm with their partner [17]. Approximately 15% of women have generally been reported to experience difficulties in reaching orgasm [18]. Higher orgasm rates are recorded for older people [17].

Lack (or loss) of sexual desire is one of the three most common sexual complaints in the general population [18], but physicians continue to be baffled about the condition. It may be proper to say that the assessment of sexual disorders [19] is a continuously evolving process. Women experiencing climacterium early are likely to perceive the problem of lack of sexual desire as a difficult issue [9], more problematic than older women do [20].

Major and minor depressive disorders are relatively common among middle-aged women, more common among women than men before the age of 55 years [21]. These disorders are commonly thought to be associated with libido and sexual activity, and some components have been suggested to be associated with aging. In a chronic pain population, two factors of the Beck Depression Inventory (BDI-21) [22] were consistently loaded: 'the physical and somatic function' and the 'negative view of the self' [23,24]. It is not known how these components are connected with sexual activity of middle-aged women.

The purpose of the present study was to examine the associations of sexual experience, orgasm experience, and lack of sexual desire with perceived health as well as the roles of negative attitude toward self, performance impairment, strenuous exercise, and menopausal symptoms as the primary explanatory variables.

## Methods

### Participants

The present investigation involved two separate cross-sectional databanks from a 15-year follow-up survey entitled the *Health and Social Support *(*HeSSup*) study. The Finnish Population Centre supplied 4 random samples stratified according to gender and age (20–24, 30–34, 40–44, and 50–54 years). The comprehensive HeSSup baseline databank of 1998 with 21,101 persons available for analysis was used for the explanatory variables. In a non-respondent analysis [25], the data were considered representative of the general population with a slight overrepresentation of women (59%).

The 2^nd ^databank entitled *Quality of Life (QoL) Among Middle-aged Women *that involved two older age groups of the HeSSup women with responses to a mail survey in 2000 was used to test outcome variables. The QoL baseline survey was mailed to (then) 42–46 and 52–56-year-old women (N = 5510) with a second mailing about two months later. A total of 3865 women responded (70% response rate after one reminder). The older group of women was more active in responding than the younger one, and women with high levels of basic and professional education in both age groups responded more often than the rest [9]. Socio-demographic background analysis of the present sexual activity variables was published in 2004 [20].

The medical ethics committee response was that because the study used a survey with "healthy" participants and did not involve hospital or clinic patients, an ethics committee approval was not necessary according to the present Finnish law. Instead, a voluntary response was adequate; the responding individuals also gave their informed consent with signature to link personal information via registries.

## Measures

### Outcome variables

The frequency of *sexual experience *(*How often are you involved in sexual interaction or otherwise experience sexual pleasure; the experience may involve sexual intercourse or something else?*) had 4 response options (*times per day*, *week*, *or month*, *more seldom*) that were grouped into 3 categories (at least once a week, at least once a month, more seldom) for analysis. Unlike many other investigations, the present study did not delimit sexual experience exclusively to intercourse – in part because sexual intercourse is not an activity equally shared among men and women: more Finnish women aged 34–74 years (7%) than men of the same age group (2%) have *never *had sexual intercourse [26]. Women (including lesbian women) having other preferences for sexual pleasure were given an equal chance to respond.

The frequency of *orgasm experience *(*How often do you experience orgasm?*) had 4 response options (*times per day*,*week*,*month*, *more seldom*) that were grouped into 3 categories (at least once a week, at least once a month, more seldom) for analysis.

*Lack of sexual desire *was solicited as one of the list of menopausal symptoms and expressed as an intensity on a continuum from 1 to 10 (1 having no lack of sexual desire at all, 10 having very severe lack of sexual desire) For the present study, four categories were used: 1 (no problem), 2–4 (slight problem), 5–7 (moderate problem), and 8–10 (severe problem).

### Primary explanatory variables

The sum of 4 other *menopausal symptoms *(*sweating, hot flashes, vaginal dryness and tenderness, sleeping problems*) was used as an explanatory variable (abbreviated SS). The menopausal symptoms were expressed as intensity on a continuum from 1 to 10 (1 having no lack of sexual desire at all, 10 having very severe lack of sexual desire) but only 4 categories were used for the present study: 1 (no problem), 2–4 (slight problem), 5–7 (moderate problem), and 8–10 (severe problem). The symptom SS was calculated by having at least 2 options, and theoretically, it had values from 2–40.

Response options of *perceived health (How is your health?*) used for the analyses were: 1=good, fairly good; 2=not good/not poor, fairly poor, or poor. Those 2 categories were used for 3 reasons: the boundaries of the extreme categories were not clear, 2 categories made the analyses easier to handle, and by combining categories, small frequencies of the extreme categories were avoided in the multivariate analyses.

*Physical exercise *was used as an example of a health activity *(How much have you exercised during your spare time or during trips to work in the last 12 months? How strenuous do you estimate the exercise to be?*). Response options included walk, brisk walk, light jogging, or brisk jogging for activity, and none. For the purposes of the present study, intensity of physical activity was estimated by using 4 optional categories (less than half an hour/week, about one hour/week, 2–3 hours/week, or 4 or more hours/week) [27]. A continuous variable was used in the analyses. For the sum, strenuous exercise was given weights.

The sub-scales of *Negative Attitudes toward Self *(*NATS*) and *Performance Impairment *(*PI*) functioned as another set of primary explanatory variables. They were created from the 21-item *Beck Depression Inventory *(*BDI*) [22]. NATS and PI were also assessed within the HeSSup study and the results paralleled with the outcomes of the study by Varjonen et al [24].

The NATS subscale included the following BDI items: mood, pessimism, sense of failure, lack of satisfaction, feelings of guilt, sense of punishment, self-dislike, self-blame, suicidal ideation, and crying. Responses with more than 3 missing items were excluded. Each item scored 0–3. The observed mean values of the NATS subscale varied between 0 and 2.90. Higher mean values reflected greater negative attitude toward self.

The PI subscale included the following BDI items: irritability, social withdrawal, indecisiveness, body image, work inhibition, sleep disturbance, fatigability, and somatic preoccupation. Responses with more than 3 missing items were excluded. Each item scored 0–3. The PI sub score comprised of the mean value of items. The observed mean values of the PI subscale varied between 0 and 2.63. Higher mean values reflected greater performance impairment.

One item of the PI subscale had to do with sexuality and was excluded. Two other items (loss of appetite, weight loss) which produced a separate factor [24] were also excluded.

### Illness indicators used for adjustments

*1. Visit to a health centre, outpatient hospital, or private physician during the last 12 months*.

*2. Physician recommended examinations or therapy*.

*3. Frequency and length of use of medications during the last year*. Hormonal replacement therapy was not asked in the baseline questionnaire of the HeSSup study. The QoL respondents were not divided into treatment & no-treatment groups.

### Statistical analyses

Univariate associations between the sexual activity variables (outcome variables) and other variables were assessed using cross-tabulations. Differences in the mean values of scores in Table 2 were tested with t-test. Multivariate associations of the outcome variables with perceived health, NATS, and PI were based on cumulative logistic regression analyses. This is the logistic regression analysis for polychotomous outcome variable measured on an ordinal scale.

**Table 1:** Frequencies of sexual and orgasm experience and of intensity of lack of sexual desire by perceived health among 42–46 and 52–56-year-old women (N = 3865)

		**Frequency of sexual experience**			**Frequency of orgasm experience**			**Lack of sexual desire**		
		1) At least once/week	Less than once/month		1) At least once/week	Less than once/month		2) Mild	Moderate	Severe
	**n**	**%**	**%**	**n**	**%**	**%**	**n**	**%**	**%**	**%**
**42–46 years Perceived health**										
poor	278	61.9	17.3	259	47.5	25.5	254	24.8	16.1	18.1
good	1363	66.5	10.1	1308	53.0	19.4	1193	31.2	11.0	6.1
**52–56-years Perceived health**										
poor	513	45.2	26.3	468	24.8	42.5	565	33.1	23.0	19.1
good	1147	54.1	20.2	1077	33.9	34.3	1226	37.4	19.0	11.8

1) Extreme categories presented

2) Extreme categories presented

**Table 2:** Means and standard deviations for main effects in the different age groups^1^

**42–46-year-old women**		**n**	**mean**	**sd**	**p-value**
**NATS**					
Perceived health	poor	316	0.50	0.51	
	good	1494	0.22	0.29	< .001
**PI**					
Perceived health	poor	316	0.63	0.42	
	good	1496	0.27	0.28	< .001
**Strenuous exercise**					
Perceived health	poor	316	27.56	26.05	
	good	1494	36.39	33.49	< .001
**Menopausal symptoms**					
Perceived health	poor	252	12.09	8.55	
	good	1194	7.89	5.60	< .001
**52–56-year-old women**					

**NATS**					
Perceived health	poor	623	0.38	0.43	
	good	1339	0.17	0.27	< .001
**PI**					
Perceived health	poor	623	0.61	0.40	
	good	1341	0.30	0.29	< .001
**Strenuous exercise**					
Perceived health	poor	619	28.48	29.59	
	good	1339	37.13	34.03	< .001
**Menopausal symptoms**					
Perceived health	poor	594	18.62	8.99	
	good	1274	15.71	8.47	< .001

^1^t-test

The associations of sexual activity with perceived health and explanatory variables of NATS, PI, strenuous exercise, and menopausal symptoms were analyzed with 6 models. The adjustment of the illness indicators (visit to physician, having health examination or therapy, use of medications) was done in all six models. In addition to the illness indicators, Model 1 included perceived health alone, Model 2 included perceived health with NATS, and Model 3 included perceived health with PI. Model 4 included perceived health with NATS and PI. Model 5 included perceived health, NATS, PI and strenuous exercise. Model 6 included perceived health with NATS, PI, strenuous exercise, and menopausal symptoms.

Associations were quantified with cumulative odds ratios (COR) with 95% confidence intervals (CI). The statistical computation was performed with the SAS system for Windows, release 8.2/2000. P-values <0.05 were interpreted as statistically significant.

## Results

All statistical analyses were performed separately for 42–46 and 52–56-year-old women. Table 1 displays the percentages of the frequencies of sexual experiences and orgasm experiences as well as lack of sexual desire expressed as an intensity of symptoms by age group and perceived health. There was a general tendency that high frequencies of sexual experiences and good perceived health coincided with each other in both age groups. Next, 42–46-year-old women reported the frequency of orgasm experiences more clearly regardless of whether they perceived their health to be good or poor. Good perceived health was equally distributed. Women who perceived their health to be good also reported mild intensity of lack of sexual desire.

Poor perceived health seemed to be associated with the experience categories more clearly among the older women and with lack of sexual desire among the younger women. Lack of sexual desire appeared about equally serious for both age groups.

Table 2 illustrates the mean scores (SD) of negative attitude toward self (NATS), performance impairment (PI), strenuous exercise, and symptoms of menopause by perceived health. Each main effect with t-test was statistically significant.

Findings from other univariate analyses have been collected into Table 3, from multivariate analyses for 42–46-year-old women into Table 4 and from multivariate analyses for 52–56-year-old women into Table 5. Except for strenuous exercise, all variables were statistically significant in the univariate analyses (Table 3).

**Table 3:** Univariate associations of sexual activity variables with analyzed explanatory variables separately for two age groups. Cumulative Odds Ratios and 95% confidence intervals for main effects (no-adjustments).

	**Frequency of sexual experience**			**Frequency of orgasm experience**			**Lack of sexual desire**		
	*Cumulative ORs for frequent sexual experience*			*Cumulative ORs for frequent orgasm experience*			*Cumulative ORs for no symptoms*		
**42–46-YEAR-OLDS**	**p-value**	**COR**	**95% CI**	**p-value**	**COR**	**95% CI**	**p-value**	**COR**	**95% CI**
**Perceived health**	0.032			0.039			<.001		
poor		1.00			1.00			1.00	
good		1.33	1.02–1.72		1.30	1.01–1.67		1.96	1.53–2.52
**NATS **^**a**^	<.001	0.78	0.71–0.86	<.001	0.79	0.72–0.87	<.001	0.75	0.68–0.82
**PI **^**a**^	<.001	0.82	0.74–0.90	<.001	0.84	0.76–0.92	<.001	0.63	0.57–0.69
**Strenuous exercise **^**a**^	0.025	1.13	1.02–1.25	0.001	1.18	1.07–1.30	0.409	1.04	0.94–1.15
**Menopausal symptoms **^**a**^	0.004	0.86	0.77–0.95	<.001	0.81	0.73–0.89	<.001	0.36	0.32–0.41
**52–56-YEAR-OLDS**									

**Perceived health**	<.001			<.001			<.001		
poor		1.00			1.00			1.00	
good		1.42	1.17–1.73		1.47	1.20–1.80		1.56	1.30–1.87
**NATS **^**a**^	<.001	0.80	0.73–0.87	<.001	0.74	0.67–0.82	<.001	0.71	0.65–0.77
**PI **^**a**^	<.001	0.82	0.75–0.90	<.001	0.75	0.68–0.83	<.001	0.64	0.59–0.70
**Strenuous exercise **^**a**^	0.273	1.05	0.96–1.16	0.053	1.09	1.00–1.20	0.869	1.01	0.93–1.10
**Menopausal symptoms **^**a**^	0.052	0.91	0.83–1.00	<.001	0.84	0.76–0.92	<.001	0.45	0.41–0.49

a. OR corresponds to change of standard deviation (sd)

**Table 4:** Associations of the sexual activity variables with analyzed explanatory variables among 42–46-year-olds. Cumulative Odds Ratios and 95% confidence intervals for the sexual activity issues for 6 models.

		**Frequency of sexual experience**			**Frequency of orgasm experience**			**Lack of sexual desire**		
		*Cumulative ORs for frequent sexual experience*			*Cumulative ORs for frequent orgasm experience*			*Cumulative ORs for no symptoms*		
***42–46 YEARS***		**p-value**	**COR**	**95% CI**	**p-value**	**COR**	**95% CI**	**p-value**	**COR**	**95% CI**
**MODEL 1**^**a**^	Perceived health	0.189			0.252			<.001		
	poor		1.00			1.00			1.00	
	good		1.21	0.91–1.60		1.17	0.89–1.54		1.74	1.32–2.28
**MODEL 2**^**a**^	Perceived health	0.708			0.712			0.006		
	poor		1.00			1.00			1.00	
	good		1.06	0.79–1.42		1.05	0.80–1.39		1.49	1.12–1.97
	NATS^b^	<.001			<.001			<.001		
			0.80	0.72–0.89		0.80	0.72–0.88		0.80	0.72–0.89
**MODEL 3**^**a**^	Perceived health	0.757			0.843			0.323		
	poor		1.00			1.00			1.00	
	good		1.05	0.78–1.42		1.03	0.77–1.37		1.16	0.86–1.56
	PI^b^	0.004			0.004			<.001		
			0.85	0.76–0.95		0.85	0.77–0.95		0.65	0.58–0.73
**MODEL 4**^**a**^	Perceived health	0.814			0.782			0.279		
	poor		1.00			1.00			1.00	
	good		1.04	0.77–1.40		1.04	0.78–1.39		1.18	0.88–1.58
	PI^b^	0.591			0.751			<.001		
			0.96	0.83–1.11		0.98	0.86–1.12		0.64	0.55–0.73
	NATS^b^	0.003			0.001			0.492		
			0.82	0.72–0.94		0.81	0.71–0.92		1.05	0.92–1.20
**MODEL 5**^**a**^	Perceived health	0.914			0.926			0.299		
	poor		1.00			1.00			1.00	
	good		1.02	0.75–1.38		1.01	0.76–1.35		1.17	0.87–1.57
	PI^b^	0.601			0.835			<.001		
			0.96	0.83–1.11		0.99	0.86–1.13		0.64	0.55–0.73
	NATS^b^	0.004			0.001			0.505		
			0.82	0.72–0.94		0.80	0.71–0.91		1.05	0.92–1.20
	Strenuous exercise^b^	0.077			0.005			0.946		
			1.10	0.99–1.22		1.15	1.04–1.28		1.00	0.90–1.10
**MODEL 6**^**a**^	Perceived health	0.583			0.502			0.556		
	poor		1.00			1.00			1.00	
	good		0.91	0.64–1.28		0.89	0.64–1.24		0.91	0.67–1.24
	PI^b^	0.569			0.925			<.001		
			1.05	0.89–1.23		1.01	0.87–1.17		0.70	0.60–0.81
	NATS^b^	<.001			0.001			0.287		
			0.74	0.64–0.86		0.78	0.68–0.90		1.08	0.94–1.24
	Strenuous exercise^b^	0.304			0.041			0.549		
			1.06	0.95–1.19		1.12	1.00–1.25		0.97	0.87–1.07
	Menopausal symptoms^b^	0.070			0.005			<.001		
			0.90	0.80–1.01		0.85	0.76–0.95		0.37	0.33–0.42

a. Adjusted for visit to physician and psychologist, having health examinations, sick leave, and life style change, use of heart medications, anti-depressants, sedatives, tranquilizers, and other medications (see methods).

b. COR corresponds to change of standard deviation (sd). COR > 1 (COR < 1) corresponds with a tendency to have more (less) frequent sexual experiences, to have more (less) frequent orgasms, or to perceive less (more) intensity of lack of sexual desire.

**Table 5:** Associations of the sexual activity variables with analyzed explanatory variables among 52–56-year-olds. Cumulative Odds Ratios and 95% confidence intervals for the sexual activity issues for 6 models

		**Frequency of sexual experience**			**Frequency of orgasm experience**			**Lack of sexual desire**		
		*Cumulative ORs for frequent sexual experience*			*Cumulative ORs for frequent orgasm experience*			*Cumulative ORs for no symptoms*		
***52–56 YEARS***		**p-value**	**COR**	**95% CI**	**p-value**	**COR**	**95% CI**	**p-value**	**COR**	**95% CI**
**MODEL 1**^**a**^	Perceived health	0.048			0.042			0.001		
	poor		1.00			1.00			1.00	
	good		1.25	1.00–1.56		1.26	1.01–1.58		1.39	1.14–1.71
**MODEL 2**^**a**^	Perceived health	0.214			0.320			0.055		
	poor		1.00			1.00			1.00	
	good		1.15	0.92–1.45		1.13	0.89–1.42		1.23	1.00–1.51
	NATS^b^	0.001			<.001			<.001		
			0.84	0.76–0.93		0.79	0.71–0.88		0.74	0.68–0.82
**MODEL 3**^**a**^	Perceived health	0.231			0.481			0.488		
	poor		1.00			1.00			1.00	
	good		1.15	0.91–1.45		1.09	0.86–1.38		1.08	0.87–1.33
	PI^b^	0.014			<.001			<.001		
			0.88	0.79–0.97		0.80	0.71–0.89		0.66	0.60–0.73
**MODEL 4**^**a**^	Perceived health	0.284			0.595			0.524		
	poor		1.00			1.00			1.00	
	good		1.13	0.90–1.43		1.07	0.84–1.35		1.07	0.87–1.33
	PI^b^	0.514			0.039			<.001		
			0.96	0.84–1.09		0.87	0.76–0.99		0.69	0.61–0.78
	NATS^b^	0.015			0.015			0.121		
			0.86	0.76–0.97		0.85	0.75–0.97		0.91	0.82–1.02
**MODEL 5**^**a**^	Perceived health	0.292			0.573			0.427		
	poor		1.00			1.00			1.00	
	good		1.13	0.90–1.43		1.07	0.84–1.36		1.09	0.88–1.35
	PI^b^	0.532			0.050			<.001		
			0.96	0.84–1.09		0.88	0.77–1.00		0.69	0.61–0.77
	NATS^b^	0.014			0.015			0.139		
			0.86	0.76–0.97		0.85	0.75–0.97		0.92	0.82–1.03
	Strenuous exercise^b^	0.725			0.368			0.221		
			1.02	0.93–1.12		1.04	0.95–1.15		0.95	0.87–1.03
**MODEL 6**^**a**^	Perceived health	0.296			0.569			0.862		
	poor		1.00			1.00			1.00	
	good		1.13	0.90–1.44		1.07	0.84–1.37		1.02	0.82–1.27
	PI^b^	0.622			0.101			<.001		
			0.97	0.85–1.10		0.89	0.78–1.02		0.76	0.67–0.85
	NATS^b^	0.016			0.026			0.112		
			0.86	0.76–0.97		0.86	0.75–0.98		0.91	0.81–1.02
	Strenuous exercise^b^	0.667			0.382			0.358		
			1.02	0.93–1.12		1.04	0.95–1.15		0.96	0.88–1.05
	Menopausal symptoms^b^	0.358			0.021			<.001		
			0.96	0.87–1.05		0.89	0.81–0.98		0.47	0.43–0.52

a. Adjusted for visit to physician and psychologist, having health examinations, sick leave, and life style change, use of heart medications, anti-depressants, sedatives, tranquilizers, and other medications (see methods).

b. COR corresponds to change of standard deviation (sd). COR > 1 (COR < 1) corresponds with a tendency to have more (less) active sex life, to perceive more (less) orgasms, or to feel less (more) lack of sexual desire.

When adjustment was done for "illness indicators" only (Model 1), good perceived health was still significantly associated with the frequency of positively-oriented sexual experience among 52–56-year-old women, orgasm experience among 52–56-year-old women, and lack of sexual desire among 42–46-year-old and 52–56-year-old women (Tables 4 and 5).

Explanatory variables of NATS and PI were statistically significant in all models in which they were included (Tables 4 and 5). Models 2–6 are complementing each other and will be examined more clearly in the next paragraphs.

## Sexual experience

Perceived health formed non-significant associations in Models 2–6.

NATS contributed negatively in close to equal strength among 42–46-year-olds and 52–56-year-olds in Models 2–5. When menopausal symptoms were added in Model 6, the association remained the same among 52–56-year-old women but became even more negative among 42–46-year-old women.

PI was statistically significant among 42–46-year-olds and 52–56-year-olds in Model 3 only.

### Orgasm experience

Perceived health formed non-significant associations in Models 2–6. NATS contributed in close to an equal strength both alone and together with other variables in Models 2–5 among 42–46-year-old women. When menopausal symptoms were added in Model 6, the association became more negative. Among 52–56-year-old women, NATS was the most negative in Model 2 but slightly increased in the positive direction in Models 3–6. PI was statistically significant both among 42–46-year-olds and 52–56-year-olds in Models 3 and 4. Strenuous exercise contributed in the significant positive vein in Models 5 and 6 among 42–46-year-old women alone. Menopausal symptoms contributed significantly among 42–46-year-olds and 52–56-year-olds.

### Lack of sexual desire

After the adjustment of using "illness indicators," the association between perceived health and lack of sexual desire (Model 1) was significant and stronger in the 42–46-year-old age group than in the 52–56-year-old one. The additional adjustment with NATS (Model 2) decreased the association more among 42–46-year-olds than among 52–56-year-olds; the association was not statistically significant. NATS was statistically significant in Model 2 only. PI also contributed negatively in close to equal strength among 42–46-year-olds and 52–56-year-olds in Models 2–5. When menopausal symptoms were added in Model 6, the association increased slightly in the positive direction both among 42–46-year-olds and 52–56-year-olds. Menopausal symptoms contributed significantly among 42–46-year-olds and 52–56-year-olds.

## Discussion

The present study indicated that the role of perceived health was relatively small in the stated three sexual issues among 42–46 and 52–56-year-old Finnish women. Statistically significant positive associations were observed in perceived health with sexual and orgasm experiences among 52–56-year-olds but not among 42–46-year-olds. As an explanatory variable, negative attitude toward self (NATS) was associated with sexual and orgasm experiences, whereas performance impairment (PI) was associated with the lack of sexual desire in both age groups. Strenuous exercise was associated with orgasm experiences in the age group of 42–46 years but not in the age group of 52–56 years. Menopausal symptoms were associated with orgasm experiences and the lack of sexual desire in both age groups.

The study was based on two older age groups of women (n = 5510) in a random sample of 21,101 individuals who responded to the initial questionnaire of the prospective follow-up survey entitled Health and Social Support (HeSSup) study. The data of the HeSSup study were representative of the Finnish population with a slight (59%) overrepresentation of women [25]. Educated women and 52–56-year-old women were more enthusiastic in responding to the present study than the rest of the women [9].

### What is left for health, when diseases and illnesses are controlled?

Self-assessed health is strongly associated with later morbidity and mortality [3], reflects health aspects not covered by other health indicators [28], and indicates a small decline with age among women but without the menopausal transition contributing to it [5]. In a summary, measures were used to determine self-reported health status and it was suggested that the outcomes of such measures reasonably well correlated with health status assessed by physician [8]. Women with good perceived health are known to visit their gynaecologists regularly [29]. Sexual or orgasm experiences (with/without a partner) are rarely encountered in outpatient gynaecological appointment settings but should be acknowledged as part of health promotion.

In the present study, 52–56-year-old women with good perceived health reported a high frequency of both sexual and orgasm experiences and *a near absence *(or a mild intensity) of lack of sexual desire, whereas 42–46-year-old women who reported good perceived health indicated a mild intensity of lack of sexual desire alone. Could it be that 42–46-year-olds will regard sexual and orgasm experiences as self-evident, whereas 52–56-year-olds are "realistic" about sex and sexuality in their age group? The older group may just perceive health as good when sexual and orgasm experiences continue to be generated at a given level.

### Negative attitude toward self (NATS) and performance impairment (PI)

NATS and PI seemed to function *as relevant components in holding subjective feelings about perceived health and as relevant explanatory factors*. An interesting finding of the present study was the manner in which NATS and PI formed associations with three outcome variables. NATS that represents the psychological variables of the Beck scale was systematically associated with sexual and orgasm experiences among both groups of women. PI that represents the somatic end of the scale is associated with lack of sexual desire among 42–46-year-olds. (PI was dissimilar among 52–56-year-olds in that it formed associations with both orgasm experiences and lack of sexual desire in a couple of the models.) In order to further define sexual health of middle-aged women, the individual issues behind NATS and PI would need to be considered more in depth. NATS or PI change from one period to another and modify the definition of sexual health. The definition of *sexual health promotion *needs to be further elucidated. The meanings of the roles NATS and PI play need to be exposed.

### Strenuous exercise

Cardiovascular endurance, muscular strength and endurance, body composition, and flexibility are among health-related components of physical fitness [30], and physical activity is demonstrated to attenuate the rate of aging- and disease-related weight loss [31]. In the present study, participation in strenuous exercise formed associations with orgasm experiences among 42–46-year-olds but not with 52–56-year-olds. Strenuous exercise is not associated with orgasm experiences among older women. Instead of strenuous activity, flexibility or cardiovascular endurance may be a more important element of fitness than muscular strength in later life. Women in their 50s tend to prefer walking, dancing, and swimming to jogging or other forms of producing an endorphin rush.

### Menopausal symptoms

In another Finnish study, most of 1308 surveyed women who turned out to be postmenopausal in a random sample of 2000 women aged 45–64 years reported good or rather good health. With the exception of hot flashes and irritability, most subjective health problems were associated with aging or something other and not with climacterium [1]. Women feel better when these events are not present and the present study confirmed the assumption.

Roughly a third or more of the woman's life span is spent following menopausal transition. This means a new existence for about 20–30 years after one has been active in personal and professional younger adult life. The number of these women will be increasing with expected life quality in those years. It is also fair to assume that many women wish to include sexual interaction of their choice as part of those years. Sexual and orgasm experiences existed among those middle-aged women who reported good or reasonably good health. In the health literature, a positive orientation of sexual and orgasm experiences should be highlighted for sexual health promotion purposes.

## Conclusion

NATS and PI are closely tied to orgasm experiences and the meaning of the roles needs to be exposed. Sexual activity deserves to be addressed more actively in patient contact at least with perimenopausal women.

## Competing interests

The author(s) declare that they have no competing interests.

## Authors' contributions

Ansa Ojanlatva and Hans Helenius conceptualized the study design together with Juha Mäkinen, and Päivi Rautava. Acquisition, analysis, and interpretation of data were made possible by Ansa Ojanlatva and Hans Helenius together with Katariina Korkeila and Päivi Rautava. Statistical analyses were performed by Hans Helenius and Jari Sundell. Drafting and critical revision of the manuscript was accomplished by Ansa Ojanlatva, Katariina Korkeila, and Päivi Rautava, and Juha Mäkinen participated in the latter. Funding was obtained by Päivi Rautava. Supervision and decision making to submit for publication were the tasks of Ansa Ojanlatva and Juha Mäkinen. All authors have read and approved the final version of the manuscript.

## References

[B1] Hemminki E, Topo P, Kangas I (1995). Experience and opinions of climacterium by Finnish women. Europ J Obstetrics & Gynecology Reproductive Biol.

[B2] Cawood EHH, Bancroft J (1996). Steroid hormones, the menopause, sexuality and well-being of women. Psychol Med.

[B3] Hunter MS, Orth-Gomer K (2003). Women's health. J Psychosom Res.

[B4] Greendale GA, Lee NP, Arriola ER (1999). The menopause. Lancet.

[B5] Dennerstein L, Dudley E, Burger H (2001). Are changes in sexual functioning during midlife due to aging or menopause?. Fertil Steril.

[B6] Stadberg E, Mattson L-Å, Milsom I (2000). Factors associated with climacteric symptoms and the use of hormone replacement therapy. Acta Obstet Gynecol Scand.

[B7] Lahdenperä M, Lummaa V, Helle S, Tremblay M, Russell AF (2004). Fitness benefits of prolonged post-reproductive lifespan in women. Science.

[B8] Heistaro S (2002). Trends and determinants of subjective health. Analyses from the national FINRISK surveys.

[B9] Jokinen K, Rautava P, Mäkinen J, Ojanlatva A, Sundell J, Helenius H (2003). Experience of climacteric symptoms among women 42–46 and 52–56 years of age. Maturitas.

[B10] Heistaro S, Vartiainen E, Puska P (1996). Trends in self-rated health in Finland 1972–92. Prev Med.

[B11] Sohlman B (2004). Functionaalinen mielenterveyden malli positiivisen mielenterveyden kuvaajana [Functional model of mental health as descriptor of positive mental health].

[B12] Dennerstein L, Dudley E, Gutrie JR (2003). Predictors of declining self – rated health during the transition to menopause. J Psychosom Res.

[B13] Kingsberg SA (2002). The impact of aging on sexual function in women and their partners. Arch Sex Behav.

[B14] Bernhard LA (2002). Sexuality and sexual health care for women. Clin Obstet Gynecol.

[B15] Kleinplatz PJ, Kaschak E, Tiefer L (2001). On the outside looking in: in search of women's sexual experience. A new view of women's sexual problems.

[B16] Davey Smith G, Frankel S, Yarnell J (1997). Sex and death: are they related? Findings from the Caerphilly cohort study. BMJ.

[B17] Mah K, Binik YM (2001). The nature of human orgasm: a critical review of major trends. Clin Psych Rev.

[B18] Lewin J, King M (1997). Editorial: Sexual medicine, towards an integrated discipline. BMJ.

[B19] Basson R, Leiblum S, Brotto L, Derogatis L, Fourcroy J, Fugl-Meyer K, Graziottin A, Heiman JR, Laan E, Meston C, Schover L, van Lankveld J, Schultz WW (2003). Definitions of women's sexual dysfunction reconsidered: advocating expansion and revision. J Psychosom Obstet Gynaecol.

[B20] Ojanlatva A, Helenius H, Jokinen K, Sundell J, Mäkinen J, Rautava P (2004). Sexual activity and background variables among women of 42–46 and 52–56 years. Am J Health Behav.

[B21] Bebbington PE, Dunn G, Jenkins R, Lewis G, Brugha T, Farrell M, Meltzer H (1998). The influence of age and sex on the prevalence of depressive conditions: report from the National Survey of Psychiatric Morbidity. Psychol Med.

[B22] Beck AT, Ward CH, Mendelson M, Mock M, Erbaugh J (1961). An inventory for measuring depression. Arch Gen Psychiatry.

[B23] Morley S, Williams AC de C, Black S (2002). A confirmatory factor analysis of the Beck Depression Inventory in chronic pain. Pain.

[B24] Varjonen J, Romanov K, Kaprio J, Heikkilä K, Koskenvuo M (1997). Self-rated depression in 12,063 middle-aged adults. Nordic J Psychiatry.

[B25] Korkeila K, Suominen S, Ahvenainen J, Ojanlatva A, Rautava P, Helenius H, Koskenvuo M (2001). Non-response and related factors in a nation-wide health survey. Eur J Epidemiol.

[B26] Haavio-Mannila E, Kontula O, Kuusi E (2001). Trends in sexual life. The Population Research Institute.

[B27] Ainsworth BE, Haskell WL, Leon AS, Jacobs DR, Montoye HJ, Sallis JF, Paffenbarger RS (1993). Compendium of physical activities: classification of energy costs of human physical activities. Med Sci Sports Exerc.

[B28] Mackenbach JP, Simon JG, Looman CW, Joung IM (2002). Self-assessed health and mortality : could psychosocial factors explain the association?. Int J Epidemiol.

[B29] Hemminki E, Sihvo S, Forsas E, Koponen P, Kosunen E, Perälä ML (1998). The role of gynecologists in women' health care – women's views. Int J Qual Health Care.

[B30] Hafen BQ, Thygeson AL, Frandsen KJ (1988). Behavioral guidelines for Health and Wellness.

[B31] Dziura J, de Leon CM, Kasl S, DiPietro L (2003). Can physical activity attenuate aging-related weight loss in older people?. Am J Epidemiol.

